# Guidelines and clinical priority setting during the COVID-19 pandemic – Norwegian doctors’ experiences

**DOI:** 10.1186/s12913-022-08582-2

**Published:** 2022-09-22

**Authors:** Berit H. Bringedal, Karin Isaksson Rø, Fredrik Bååthe, Ingrid Miljeteig, Morten Magelssen

**Affiliations:** 1 Institute for Studies of the Medical Profession, Oslo, Norway; 2 Institute of Stress Medicine - ISM at Region VGR, Gothenburg, Sweden; 3 Institute of Health and Care Sciences, Sahlgrenska Academy, Gothenburg University, Gothenburg, Sweden; 4 Bergen Centre for Ethics and Priority Setting (BCEPS), Department of Global Public Health and Primary Care, University of Bergen, Bergen, Norway; 5 Department of Research and Development, Helse Bergen Health Trust, Bergen, Norway; 6 Centre for Medical Ethics, Institute of Health and Society, University of Oslo, Oslo, Norway; 7 MF Norwegian School of Theology, Religion and Society, Oslo, Norway

**Keywords:** COVID-19, Clinical priority-setting, Guidelines, Priority-setting, Rationing

## Abstract

**Background:**

In the first phase of the COVID-19 pandemic, strong measures were taken to avoid anticipated pressure on health care, and this involved new priorities between patient groups and changing working conditions for clinical personnel. We studied how doctors experienced this situation. Our focus was their knowledge about and adherence to general and COVID-19 specific guidelines and regulations on priority setting, and whether actual priorities were considered acceptable.

**Methods:**

In December 2020, 2 316 members of a representative panel of doctors practicing in Norway received a questionnaire. The questions were designed to consider a set of hypotheses about priority setting and guidelines. The focus was on the period between March and December 2020. Responses were analyzed with descriptive statistics and regression analyses.

**Results:**

In total, 1 617 (70%) responded. A majority were familiar with the priority criteria, though not the legislation on priority setting. A majority had not used guidelines for priority setting in the first period of the pandemic. 60.5% reported that some of their patients were deprioritized for treatment. Of these, 47.5% considered it medically indefensible to some/a large extent. Although general practitioners (GPs) and hospital doctors experienced deprioritizations equally often, more GPs considered it medically indefensible. More doctors in managerial positions were familiar with the guidelines.

**Conclusions:**

Most doctors did not use priority guidelines in this period. They experienced, however, that some of their patients were deprioritized, which was considered medically indefensible by many. This might be explained by a negative reaction to the externally imposed requirements for rationing, while observing that vulnerable patients were deprioritized. Another interpretation is that they judged the rationing to have gone too far, or that they found it hard to accept rationing of care in general. Priority guidelines can be useful measures for securing fair and reasonable priorities. However, if the priority setting in clinical practice is to proceed in accordance with priority-setting principles and guidelines, the guidelines must be translated into a clinically relevant context and doctors’ familiarity with them must improve.

**Supplementary Information:**

The online version contains supplementary material available at 10.1186/s12913-022-08582-2.

## Background

The first phase of the COVID-19 pandemic involved uncertainty and pressures on patients and staff in the health care system. Fearing the conditions seen in Italy and Spain in March 2020, various measures were taken in Norway, to avoid unmanageable pressure on hospital beds [1]. Reallocation of staff and preparation of the health care system for the potential wave of infected patients, together with strict quarantine regulations, led to a scarcity of staff and many patients did not receive the care they would have received in a normal situation [1, 2].

The crisis strongly accentuated the need to prioritize resources. Although priority setting guidelines and regulations already were in place in several countries, Norway included, a much stronger strain on the health services than normal was anticipated. Hence, the health authorities in many countries issued specific priority guidelines adjusted to the (predicted) forthcoming crisis, in particular for, but not limited to, triage in intensive care units [3].

Our research group examined the various aspects of how Norwegian doctors experienced the situation during the first period of the pandemic (between March and December 2020). Hence, the current paper focuses on how they experienced prioritization between patient groups and guidelines for priorities.

### *The Norwegian health care system* [4]

The health care system is a single payer, universal coverage system, primarily funded by general taxes. General budgets are allocated by the government. Hospital care is organized as regional trusts with independent boards. Yearly contracts are made between the trusts and Ministry of Health, which decides the total budget and expected quantum and quality of care. Primary care is locally organized, by the municipalities, and GPs are organized as private businesses who receive public funding based on capitation (number of patients on their lists), number of consultations and procedures (e.g., lab, ultrasound), and patient co-payments. GPs are the gatekeepers of specialist care.

### Priority setting regulations and guidelines

Norway has a relatively long history of priority setting initiatives in health care [5]. The first governmental committee was appointed in 1987 [5], and since then, a number of initiatives have been carried out. Today, three priority setting criteria have been approved by the Norwegian parliament. The *utility criterion* states that the priority of an intervention increases with its expected health benefits; the *resource criterion* states that the priority of an intervention increases the less resources it requires; and the *severity criterion* states that the priority of an intervention increases with the severity of the condition in question [6, 7].

The criteria are implemented in two main pieces of legislation. Firstly, the Specialist Health Services Act regulates specialized health services (somatic and mental care), both inpatient and outpatient care. In this act, § 2–1 a, which was amended in 2019, requires regional health trusts to organize their services in line with the three criteria. Second, the Priority Regulation [8] emphasizes the first two priority setting criteria by stating that the allocation of resources should be made according to the expected health benefit from the intervention, while the costs should be reasonable in relation to its expected benefit.

In a white paper approved by parliament in April 2022, the government proposed the implementation of the same three priority setting criteria for primary health and care services as well, with one amendment: *Patient coping* should also be considered when judging utility of treatment and severity of disease [7].

In addition to these generally applicable regulations, the Norwegian Directorate for Health soon issued specific guidelines to be applied in the pandemic, giving advice, for example, on prioritization in nursing homes and home-based care and the rationing of care in intensive care units [9].

A fundamental tenet of Norwegian health law is that “health personnel must carry out their work in accordance with requirements of professional soundness and care” (Health Personnel Act § 4). A key medico-legal requirement is the health care system's responsibility to avoid "medically indefensible"/”professionally unsound” (in Norwegian: "*faglig uforsvarlig*") care. The term refers to care that is deficient in such a way that legal liability might be incurred for the practitioner and/or the institution. Thus, “medically indefensible” points to a crucial minimal standard or threshold against which the quality of care must be judged. Against this background, we included the question of whether the doctors considered any deprioritization as medically indefensible.

Although the health authorities have put much energy into issuing guidelines on priority setting, it is quite another question whether health professionals follow them. Priority setting legislation have been put in place since the beginning of 1990. Since then, a number of committees, white papers, and more legislation are put in place, while their effects on actual prioritization of resources in health care should be further researched.

Most likely, health authorities assume that legislation and guidelines are known and followed by health care workers; this must be considered the rationale behind issuing guidelines in the first place. It is, however, not self-evident that guidelines are followed [10–12]. Hence, in the current study, we asked the doctors about their knowledge of priority setting rules and guidelines, to what extent they used them during the beginning of the pandemic, and whether they considered the criteria and guidelines reasonable. We focused on how they experienced the actual priorities that were made, if there were changes in the priorities between patients, whether certain patient groups were deprioritized, and to what extent this was seen as medically defensible.

### Hypotheses

Our preconception was that doctors have limited knowledge of the national priority guidelines – both general and pandemic-specific ones - and that most doctors do not use them, neither in normal circumstances nor during the pandemic. We further assumed that they had to make, or were faced with, some hard priorities during the pandemic with which they did not always agree. Finally, we assumed that there were systematic differences in their views depending on their workplace.

From the fact that mental health care often has lost out to somatic care in the competition of medical resources, we suspected that psychiatrists had experienced lower priority of their patients more often than others.

Based on these preconceptions, we formulated nine hypotheses about (i) familiarity with and use of priority setting criteria and guidelines, and (ii) experiences with actual priorities during the pandemic (Table 1).

**Table 1:** Nine hypotheses about doctors’ experiences with priority setting during the pandemic

Topic	#	Hypothesis
(i) Familiarity with priority setting criteria and guidelines	H1	A majority of doctors were familiar with the three official priority setting criteria
	H2	A majority of doctors were *not* familiar with the legislation concerning priority setting
	H3	A majority of doctors were *not* familiar with the national guidelines for priorities during the pandemic
	H4	More doctors in managerial positions were familiar with the guidelines
	H5	A majority of doctors did *not* use the national guidelines for priorities during the pandemic
(ii) Experiences with actual priorities during the pandemic	H6	A majority of doctors experienced that some of their patients were given a lower priority
	H7	Of those who experienced lower priority for their patients, a majority deemed this medically indefensible
	H8	Doctors in psychiatry more often reported patients being given a lower priority than doctors in somatic care
	H9	Doctors in primary care (i.e., GPs, nursing home doctors) more often reported their patients being given lower priority than doctors in hospitals

## Methods

### Data

The data were extracted from the 2020/2021 dispatch of a survey of a representative sample of doctors working in Norway (the Norwegian Physician Survey), which is sent out every second year. The panel was established in 1994, and has been complemented six times thereafter with new, younger members. Only working doctors are part of the panel. Its representativeness is compared with the Norwegian Medical Association’s (NMA) list of members. According to the NMA, the list includes more than 90% of all doctors in Norway. The panel shows the same distribution as the population in terms of age, gender, and proportions of those doctors working in primary care, hospitals, and private specialist care.

In December 2020, a questionnaire was sent to the 2 316 members of the panel, with two reminders in the first months of 2021. The questionnaire covered various experiences during the pandemic, including working conditions, time pressure, access to personal protective equipment, moral stress, and priority setting. We also included a question that sought answers in a free text format, as follows: "If some of your patients received lower priority, which patient groups did this concern?" Answers were independently interpreted, coded and grouped by two of the authors.

In addition to questions specifically related to the pandemic, the questionnaire also included questions about work, stress and professional satisfaction, the primary care doctors' role in treatment for depression, and patient lifestyle and priority to care.

The questions relevant to the present article have been translated into English and are available in Supplementary File 1.

### Ethics approval

According to the Regional Committee for Medical Research Ethics, a study based on "The Norwegian Physician Survey—A prospective questionnaire survey of a representative sample of Norwegian physicians" is exempt from review in Norway, cf. §§ 4 of The Act. Thus, the current project could thus be implemented without the approval of the Regional Committee for Medical Research Ethics (IRB 0000 1870). In addition, approval for data protection of the prospective survey among Norwegian doctors was obtained from the Norwegian Centre for Research Data (NSD, reference 19,521). All participants gave their written consent.

### Analysis

The data were analyzed with descriptive statistics and logistic regression analyses. priority setting Although we did not perform traditional hypothesis testing, we formulated our preconceptions as hypotheses (Table 1). The hypotheses about differences between the subgroups of doctors were analyzed either by non-overlapping 95% Confidence Intervals (CI), cross-tabulation and chi square or by logistic regression. The variables included were age, gender, specialty (psychiatry or not), and workplace (general practice or hospital practice). Categorical variables were dichotomized in the logistic regression analyses. Significance level: *p* < 0.05. All methods were carried out in accordance with the relevant guidelines.

## Results

### Knowledge and application of priority setting criteria and guidelines

When asked whether the doctors were familiar with the three official priority setting criteria, 62.5% responded "yes". 16.4% reported familiarity with the general priority setting legislation. Knowledge about the contents of the Health Directorate's specific priority guidelines for COVID-19 [9] was also scarce; 21.6% reported knowing its contents. (See footnote * in Table 2 explaining the coding of these variables.)

**Table 2:** Percentages of doctors who are familiar with the guidelines on priority setting. Percentages and (95% CI)

Are you familiar with:	Doctors in managerial positions (*n* = 125–127)*	Senior hospital doctors (*n* = 402–405)*	General practitioners (*n* = 282–290)*	Doctors in specialization, private specialists, others (*n* = 693–696)*	N
The three official priority setting criteria?^i^	**83.5%**^**a**^ (77.0–90.0)	63% (58.3–67.7)	63.1% (57.6–68.7)	59% (55.3–62.7)	1513
The legal regulation for priority setting^ii^ ("Prioriterings-forskriften")	**42.4%**^**b**^ (33.7–51.1)	**23.6%**^**b**^ (19.5–27.8)	9.6% (6.2–13.0)	10.2% (8.0–12.5)	1503
The guidelines for the COVID-19-pandemic^iii^	**50%**^**a**^ (41.3–58.7)	23% (18.9–27.1)	21.7% (17.0–26.4)	15.5% (12.8–18.2)	1517

^*^The number answering varied according to which regulation/guideline was asked about

^i^Response alternatives "yes" and "no"

^ii^Response alternatives dichotomized into yes (including "yes" and "to some degree") and no^i^

^iii^Response alternatives dichotomized into "yes and know its contents", and no (including "yes" (but do not know the contents and "no")

^a^Bold figures indicate that doctors in managerial position had a significantly higher percentage answering yes than doctors in the other three categories (estimated through nonoverlapping 95% CI)

^**b**^^b^Bold figures indicate that senior hospital doctors and doctors in managerial positions had a significantly higher percentage answering yes than for doctors in the other two categories (estimated through nonoverlapping 95% CI)

We hypothesized that there would be differences in familiarity depending on the position. As shown in Table 2, more doctors in managerial positions were familiar with all the regulations and guidelines. When controlling for age and gender, the pattern was the same (data not shown).

When asked if the doctors had been using any priority setting guidelines, a majority answered that they had not (Table 3). The most commonly used priority setting guidelines were those for the respondents' specific department or practice.

### Actual priorities during the pandemic

**Fig. 1:**
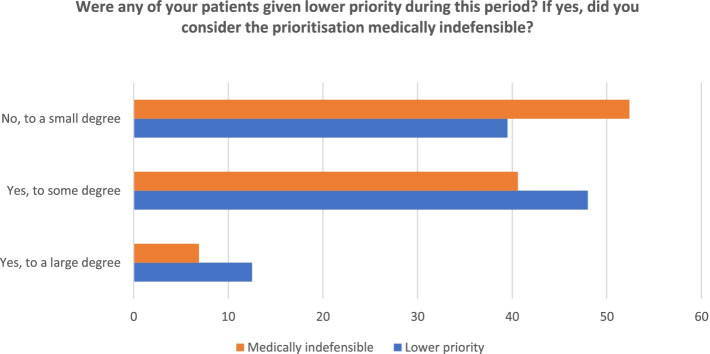
Agreement to statements about lower priority. Percentages. N/A excluded

We assumed that many doctors more often had to prioritize between patients during the pandemic and that some of their patients were given a lower priority. We further hypothesized that some of these priorities were considered contrary to what the doctors judged as medically defensible. The responses are shown in Fig. 1. A majority had experienced that their patients had received lower priority because of the pandemic, either to some (48%) or a large degree (12.5%, in total 60.5%). Almost half of those who experienced deprioritization of some of their patients judged this to have been medically indefensible to some (40.6%) or to a large degree (6.9%).

We assumed that the doctors had different experiences depending on their specialty and workplace. The data showed, however, that this was not the case. 54.7% of psychiatrists had experienced lower priority of their patients vs 61.2% among somatic doctors (Chi square 2.5, *p* = 0.11). 60.1% of GPs (including nursing home doctors) reported that their patients had been given lower priority, vs 62.5% among hospital doctors (Chi square 0.48, *p* = 0.49).

A logistic regression analysis of the effect of workplace (hospital vs primary care), age and gender showed, on the other hand, an age effect. The probability of reporting that their patients had been given lower priority increased with lower age (Exp (B) = 0.97, 95% CI = 0.96–0.98, *p* < 0.001, when controlling for workplace and gender. See Supplementary File 2.

Among those who partly or fully agreed that they had seen a lower priority for their patients, 404 of 849 (47.5%) considered these priorities to be medically indefensible. A logistic regression analysis of the effect of age, gender and workplace, showed no age effect, but doctors working in primary care were significantly more likely to consider the priorities as medically indefensible (Exp (B) = 1.87, 95% CI = 1.32–2.65, *p* < 0.001). See Supplementary File 2.

The question "If some of your patients received lower priority, which patient groups did this concern?" was answered by 407 respondents. Many listed more than one patient group. The three most common were patients with psychiatric diagnoses and addiction (*n* = 65), patients in need of ' treatment and follow up consultations (*n* = 55), and patients with chronic diseases/multimorbidity and high age (*n* = 30).

Table 4 summarizes what we found compared with what we hypothesized.

**Table 3:** Responses to the question: “Did you use any of these guidelines during the pandemic?”

Guideline/regulation	No % (95% CI)	Yes % (95% CI)	N*
Priority setting guidelines for my hospital/my municipality	65.7 (62.1–69.3)	34.3 (29.3–39.3)	996
Priority setting guidelines for my department/my practice	60.9 (57.0–64.8)	39.1 (34.2–44.0)	984
The priority regulation	75.2 (72.1–78.3)	24.8 (19.4–30.2)	993
The regulation of specialist services	68.9 (65.4–72.4)	31.1(26.0–36.2)	1000

*Doctors in non-clinical positions and those responding N/A were excluded

**Table 4:** Nine hypotheses and whether they were confirmed

Topic	#	Hypothesis	Confirmed?
(i) Familiarity with priority setting criteria and guidelines	H1	A majority of doctors were familiar with the three official priority setting criteria	Yes
	H2	A majority of doctors were *not* familiar with the legislation concerning priority setting	Yes
	H3	A majority of doctors were *not* familiar with the national guidelines for priorities during the pandemic	Yes
	H4	More doctors in managerial positions were familiar with the guidelines	Yes
	H5	A majority of doctors did *not* use the national guidelines for priorities during the pandemic	Yes (partly)
(ii) Experiences with actual priorities during the pandemic	H6	A majority of doctors experienced that some of their patients were given a lower priority	Yes
	H7	Of those who experienced lower priority for their patients, a majority deemed this medically indefensible	Yes
	H8	Doctors in psychiatry more often reported patients being given a lower priority than doctors in somatic care	No
	H9	Doctors in primary care (i.e., GPs, nursing home doctors) more often reported their patients being given lower priority than doctors in hospitals	No

## Discussion

A majority of the doctors in the current study reported familiarity with the general criteria for priority setting and, to some extent, the priority setting legislation ("Prioriteringsforskriften"), while, at the same time, being unfamiliar with the specific guidelines for priority setting under the pandemic. A large majority (between 60.9% and 75.2%, depending on the guideline) had not used any guideline at all under the pandemic. Further, most reported having seen changes in priorities between patient groups and that some of their patients were deprioritized. Finally, 47.5% considered these priorities medically indefensible to some (40.6%) or a large (6.9%) degree, and GPs most often did so.

### Insufficient knowledge of priority setting guidelines

These findings tell us, first, that the clinicians' knowledge and use of specific guidelines for priority setting in the clinic is rather scant. This is remarkable because the health authorities presumably issue guidelines with the expectation that they be used generally and in extraordinary situations. For the health authorities in a country that has debated, adopted and implemented priority setting principles and guidelines for decades, this is a finding that should attract attention. Clearly, adopting principles and enacting legislation is insufficient for priority setting principles to be consciously utilized by clinicians in their daily work.

Although Norway has well-established general priority setting criteria (viz. utility, resource use and severity), the health authorities clearly considered them too general to be sufficiently action-guiding during the pandemic. Hence, the need arose for more specific, tailored priority setting advice. In the Norwegian Directorate of Health’s priority setting guideline for the pandemic [9], the degree of explicitness and concreteness varied between fields. For example, in the case of ICU, the priority guidelines were explicitly built on the priority criteria and provided concrete priorities, while the priority guidelines for the primary health and care services did not explicitly refer to the criteria.

The general criteria are not only too general in a pandemic situation, but need to be operationalized for specific contexts also in normal times. This has been acknowledged by health authorities, and priority setting guides were established for 33 medical specialties between 2008 and 2012 [13]. The guides aid Norwegian hospital clinicians in evaluating referrals to their departments. They explicitly build on the three priority setting criteria. Developing similar guides for other areas of Norwegian healthcare would be an interesting prospect which would be one way of bringing the priority setting criteria out to clinicians.

Our findings of insufficient knowledge and usage of priority setting principles and legislation corroborate the findings in a 2018 Directorate of Health report [14]. The authors of the report urged that clinicians be taught priority setting theory and how such theory can be applied in practical work; and that venues for multidisciplinary reflection and discussion on priority setting issues be established.

Clinicians are used to and sympathetic to clinical guidelines. A Norwegian study found that clinical guidelines belonged to the least controversial of the instruments aimed at governing clinical care [15]. A study of physicians and nurses in Western Norway in 2020, found that most of the 1606 participants agreed with national and local prioritization guidelines. Here, 83% and 80% agreed or strongly agreed, while 5% disagreed and 7% disagreed strongly [2].

However, the actual use of clinical guidelines seems to vary considerably [16], which has been confirmed in our study. If priority setting in clinical practice is to proceed in accordance with the priority setting principles and guidelines, these must be translated into a clinically relevant context, and doctors’ familiarity with them must improve.

### Reluctance to rationing care

Priority setting guidelines are different from clinical guidelines. Whereas the aim of a clinical guideline is to provide the doctor with the best evidence for making decisions, that is, to contribute to the best diagnostics or treatment for improving the patient's health, priority guidelines are explicitly limit setting. This potentially makes the latter harder to adhere to. Although it can be argued that clinical guidelines indirectly set limits as well, the priority guidelines aim solely at rank ordering treatments, hence also ranking patients. This is a much harder and ethically more controversial undertaking.

The scarcer the resources, the harder the priority setting. Under normal circumstances, clinicians find it hard to prioritize; in a crisis, the no's become even more evident, frequent, and potentially controversial. For example, the Italian guidelines [17] were criticized for ageism [3], because it was explicitly stated that an age limit "may ultimately need to be set". In our study, many doctors expressed concerns about the priorities that were made, and an impression of systematic deprioritization of certain patient groups (e.g. older patients) might have contributed to this judgment.

Many doctors, as well as leaders and politicians, find it hard to say no. The doctor's traditional duty is to give the patient good care, not to deny care. This might be an even stronger predicament for doctors if they perceive that such a rationing has not been officially and explicitly mandated. Arguably, the social contract of which healthcare is part involves the expectation that every patient will receive care of sufficient quality. This is especially true in a situation in which the need for significant rationing has not been officially acknowledged, and in which having to ration in such a way is likely to lead to moral distress for doctors.

Furthermore, there is an inherent tension between the roles for the clinician, as the patient's advocate on the one hand, and the responsibility to allocate resources in line with the general priority criteria on the other hand [18]. This can lead to unjustified inequalities between patient groups if the power to influence resource allocation varies between specialties.

### Medically indefensible priorities

Not only did we find that the clinicians' knowledge and use of priority guidelines was limited, but our findings also indicate that even though the priority guidelines were not applied, the pandemic still involved a change in priorities, many of which were considered medically indefensible. It is not clear how the concept "medically indefensible" was interpreted by the doctors in the study, or if it was interpreted similarly by all the respondents. Whether they considered the rationing to be indefensible in strict legal terms—involving liability—is not clear. Our interpretation is that a concern was expressed; that the rationing was considered problematic from a medical viewpoint. However, the study was not designed to shed light on the severity of the actual consequences for the patients in question.

Why did the decisions about priorities change? Our data cannot tell us this, but we know that it was not (mainly) a result of the individual doctor's use of the priority setting guidelines, at least according to their own reports. If the clinicians did not implement the priority guidelines, the decisions on changing priorities must have been made by others. In a hospital setting, the hierarchical system could indicate that priority decisions are made by the department and/or hospital leaders. This could relieve individual doctors from the responsibility of deprioritizing their own patients, but increase the experience of imposed prioritizations and make it psychologically more probable to be critical of the decisions. We saw no difference between hospital doctors and GPs regarding the experience of deprioritization, while more GPs considered the priorities to be medically indefensible. A possible reason for this may be that the GPs were closer to the nursing homes, where the mortality rates were the highest [19].

It is, however, unreasonable to believe that considering priorities as medically indefensible can be explained solely by a lack of co-decisioning. It was reported that the patient groups that suffered the most were the same patient groups who notoriously come out behind the others in more normal times, namely the chronically ill, psychiatric patients, people with addictions, and patients with co-morbidities (often high age). Observing that the normally lowest prioritized patients became even less prioritized in the pandemic, could be a reasonable cause for concern, perhaps even moving some to consider the priorities as a potential breach of law.

Another aspect of this pattern is that the authorities' demand to free up capacity through postponing elective surgery likely exacerbated existing social inequalities. A study of the NHS [20], concluded that "*As ever, those individuals, and countries with least resources, were affected most by the shock.*" Studies of previous pandemics supports the conclusion [20], and there is no reason to assume that Norway differs from other countries in this respect. Because the distribution of health and illness follows a social gradient [21], the disruption of elective treatment will hit the various patient groups in a socially skewed pattern.

Guidelines can be a valuable aid when allocating scarce resources. They suggest that the hard decisions on limits to care are moved away from the doctor-patient relation, which can relieve tensions between the two. Guidelines can also reduce some tensions between the doctors' different roles, ideally also helping reduce the unjustified inequalities between patient groups. This requires however, that the guidelines be based on good evidence and common values, that clinical decision makers accept and use them, and that there are effective and transparent systems for changes in, and evaluations of, the guidelines. As we have seen in the present study, the guidelines were not used by the individual clinician, and the priority decisions that were made were to a large extent considered indefensible. This should be important knowledge for health authorities.

### Strengths and weaknesses

A strength of the current study is the closeness in time between the start of the COVID-19 pandemic and the measurements asking for the doctors´ experiences and views. This reduces the potential for false memories. It should be acknowledged, however, that the pandemic is not over. The patterns we have seen here might change over time. Clearly, we cannot know whether the findings pertain only to the initial phase.

The relatively high response rate (70%) is another strength. This is higher than for other surveys of the medical profession [22]. The sample is also representative of the population of practicing doctors in Norway, in key aspects like gender, age and workplace [23]. This provides a good basis for generalization, but does not entirely rule out the possibility of nonresponse bias.

Because the pandemic laid a large amount of stress on health personnel, it is possible that the most stressed doctors opted out of answering the questionnaire. On the other hand, doctors who were confronted with harsh priorities might have a stronger interest in reporting back on their experiences.

Another limitation is that we only have self-reported data. When investigating attitudes, this is a plausible methodology. However, there is much more uncertainty when it comes to self-reports on actual behavior. The responses to the question of which patients groups suffered the most is, however, substantiated by other studies of systematic deprioritizations in health care.

We have not used validated questions because we could not find any good examples that explore what we wanted to study.

## Conclusion

If priority setting in clinical practice is to proceed in accordance with priority setting principles and guidelines, doctors’ familiarity with, and use of, these principles and guidelines must improve. Apparently, practical priority setting in response to the pandemic was considered medically indefensible by many doctors. One interpretation is that doctors have judged that the rationing of care goes too far. Another interpretation is that society in general, including politicians, patients, and doctors, often finds it hard to accept rationing of care for particular patient groups.

## Supplementary information

**Additional file 1.** Excerpts from the questionnaire.

**Additional file 2.** Logistic regression analysis of experiences of lower priority and judgements of medically indefensibility controlled for age, gender and workplace.

## Data Availability

The datasets for this study are not publicly available because of legal and ethical restrictions (the participants did not agree for their data to be shared publicly) but are available from the corresponding author upon reasonable request.
