# Supplementary material for: Guidelines and clinical priority setting during the COVID-19 pandemic – Norwegian doctors’ experiences
Source: BMC Health Serv Res. 2022 Sep 22;22:1192. doi: 10.1186/s12913-022-08582-2 (PMC9503249; doi:10.1186/s12913-022-08582-2)
Supplement: Supplementary file 1 — Additional file 1. Excerpts from the questionnaire. [file 12913_2022_8582_MOESM1_ESM.docx]

***Supplementary file 1: Excerpts from the questionnaire***

| **B04** |  | No/to a little extent | Yes, to some extent | Yes, to a large extent | Not applicable |
| --- | --- | --- | --- | --- | --- |
| B04.01 | Were any of your normal patients groups given lower priority during the pandemic? | 1 | 2 | 3 | 9 |
| B04.02 | If yes, do you find the deprioritization indefensible? | 1 | 2 | 3 | 9 |

| B04.03 | If yes, what patient groups suffered the most? |
| --- | --- |
|  | Please specify: ….. |

| **B05** |  | Yes, and know its contents | Yes, but not its contents | No |
| --- | --- | --- | --- | --- |
| B05.01 | Are you familiar with the Directorate of Health's "Priority setting in Norwegian health care during the covid-19 pandemic" ? | 1 | 2 | 3 |

| **B06** | **Did you use any of these guidelines during the pandemic?** | No | Yes | Not applicable |
| --- | --- | --- | --- | --- |
| B06.01 | Guidelines for priority setting in my hospital/organisation/ municipality | 1 | 2 | 9 |
| B06.02 | Guidelines for priority setting in my department/practice | 1 | 2 | 9 |
| B06.03 | The priority setting legal regulation ("Prioriteringsforskriften") | 1 | 2 | 9 |
| B06.04 | The Specialist Health Services Act | 1 | 2 | 9 |
| B06.05 | None specific | 1 | 2 | 9 |
| B06.06 | Other guidelines: … | 1 | 2 | 9 |

| **B07** |  | No | Yes |
| --- | --- | --- | --- |
| B07.01 | **Are you familiar with the three official criteria for priority setting, "benefit", "severity", and "use of resources»?** | 1 | 2 |

| **B08** |  | No | To some extent | Yes |
| --- | --- | --- | --- | --- |
| B08.01 | **Are you familiar with the priority setting regulation?** | 1 | 2 | 3 |
| B08.02 | **If yes, do you think it includes the main factors to consider in a priority setting situation?** | 1 | 2 | 3 |
