# Supplementary material for: Guidelines and clinical priority setting during the COVID-19 pandemic – Norwegian doctors’ experiences
Source: BMC Health Serv Res. 2022 Sep 22;22:1192. doi: 10.1186/s12913-022-08582-2 (PMC9503249; doi:10.1186/s12913-022-08582-2)
Supplement: Supplementary file 2 — Additional file 2. Logistic regression analysis of experiences of lower priority and judgements of medically indefensibility controlled for age, gender and workplace. [file 12913_2022_8582_MOESM2_ESM.docx]

**Supplementary File 2**

**Logistic regression analysis of experiences of lower priority and judgements of medically indefensibility controlled for age, gender and workplace**

*Age: Continuous*

*Gender: Reference category Male 0 , Female 1*

*Workplace: Reference category , Hospital 0, Primary care 1*

**Were any of your patients given lower priority during this period?**

**Logistic regression analysis controlling for Age, gender and workplace**


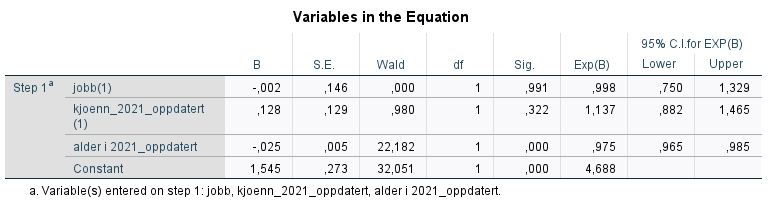


**If lower priority of your patients, did you consider this medically indefensible?**

**Logistic regression analysis controlling for Age, gender and workplace**

**
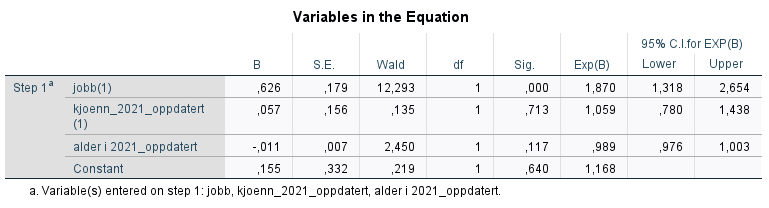
**
